# Immunoprophylactic Potential of a Cocktail of Three Low Molecular Weight Antigens of *Leishmania donovani* along with Various Adjuvants Against Experimental Visceral leishmaniasis

**Published:** 2018

**Authors:** Harpreet KAUR, Ankita THAKUR, Sukhbir KAUR

**Affiliations:** Parasitology Laboratory, Department of Zoology, Panjab University, Chandigarh, 160014, India

**Keywords:** Cocktail antigens, Experimental visceral leishmaniasis, Adjuvants

## Abstract

**Background:**

Currently, there is no vaccine available for any form of leishmaniasis for human use, including visceral leishmaniasis (VL). The treatment relies on drugs associated with severe toxic side effects and increased parasite drug resistance. At present, there is a strong need to develop and implement a successful vaccine against this disease. Therefore, we evaluated immunoprophylactic potential of a cocktail of low molecular weight antigens along with various adjuvants.

**Methods:**

The three antigens (2015, Department of Zoology, Panjab University, Chandigarh), 31kDa, 36 kDa and 51 kDa of *L. donovani* were used in this study. Inbred BALB/c mice were immunized with 10 μg of cocktail antigens i.e. 31+36+51kDa alone and along with different adjuvants (ALD, saponin, and liposome). Mice were boosted twice at an interval of 2 wk and after last dose; mice were given challenge infection with 107 promastigotes. Mice have sacrificed15 d post immunization and on 30, 60, 90 post-challenge days for evaluation of different parameters.

**Results:**

Immunized animals showed reduced parasite load, increased DTH responses and elevated levels of IgG2a antibody. The levels of Th1 cytokines were higher as compared to Th2 cytokines in immunized animals.

**Conclusion:**

Best results were obtained with cocktail of 31+36+51+liposome and this combination conferred maximum protection.

## Introduction

Visceral leishmaniasis is caused by dimorphic protozoan parasites of genus *Leishmania*. Every year 200000 to 400000 new cases of VL occur (1). In the endemic regions of visceral leishmaniasis of the world, the situation has complicated due to high toxicity and increasing drug resistance. Immunization of animals with defined subunit vaccines or live-attenuated strains and killed vaccines of Leishmania can induce significant protection (2). Although substantial efforts have been made by many laboratories, no such vaccine is available until date. The clinical trials of first-generation vaccines in humans have assessed the effect of three types of vaccines (3). Most of the vaccine studies in past have been performed using second generation vaccine candidates like GP36, 31 kDa and 32 kDa (4).

Numerous studies have been conducted with various antigens in vaccine research against leishmaniasis. The use of FML as the antigenic molecule has been described previously (5). It is a 36-kDa glycoprotein present in both stages of *Leishmania* parasites. The use of this antigen in serological diagnosis has resulted in 100% sensitivity and 96% specificity. The 31kDa protein of *Leishmania* promastigote has been used in diagnosis. In a study, the 31-kDa polypeptide was identified by 100% of the serum samples from VL patients and 73%–100% of the serum samples from patients cured of VL (6). The 51 kDa *Leishmania* protein has also been identified in 30% of blood samples from VL patients (7). Besides, this 51 kDa antigen has been tested for PCR based diagnosis of VL (8, 26)

An effective antileishmanial vaccine cannot consist of a single antigen (2).Therefore, cocktail vaccines comprised of multiple antigens along with suitable adjuvants have more chances to become successful. Adjuvants when added to antigens they boost the immune response. ALD, saponin and liposomes have previously been used as an adjuvant in different laboratories and have shown promising results (9,10). So, the present study was planned with a cocktail of 31+36+51kDa antigens of *L. donovani* along with ALD (Autoclaved *L. donovani*), saponin and cationic liposomes against murine visceral leishmaniasis.

## Materials and Methods

The study was conducted in Department of Zoology, Panjab University, Chandigarh, India in 2015.

**Parasite:** The promastigotes of *L. donovani* of strain MHOM/IN/80/Dd8 were used. The promastigotes were subcultured after every 48-72hin NNN media supplemented with MEM.

**Animals:** Inbred BALB/c mice of either sex were procured from Central Animal House, Panjab University, Chandigarh. They were fed with water and mouse feed ad libitum.

### Identification and electro-elution of antigens

**Fig. 1:**
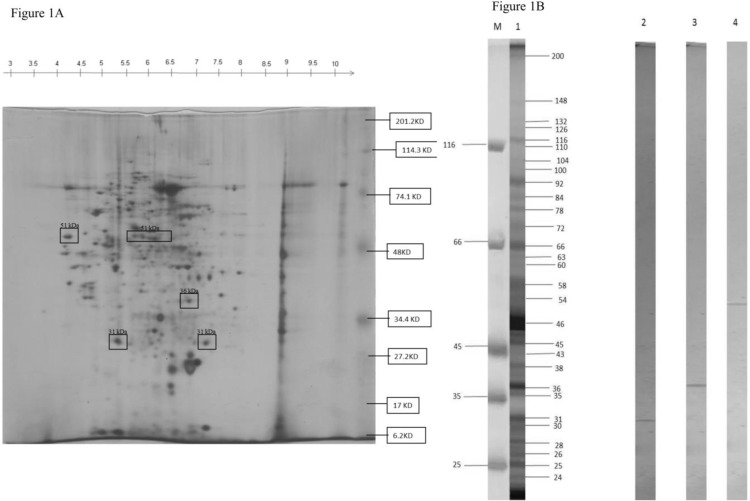
**(A)** 2-D gel electrophoresis of promastigote antigens of *L. donovani* pH 3–10: Immobiline strip of pH 3–10 was used in 1-D isoelectric focusing **(B)** SDS-PAGE of promastigote antigens of *L. donovani* and eluted 31, 36 and 51kDa antigens Lane M: molecular weight markers, lane 1: promastigote antigens, lane 2: eluted 31kDa antigen, lane 3: eluted 36kDa antigen, lane 3: eluted 51kDa antigen

The antigens were identified using 2-D gel electrophoresis using molecular weight markers (Fig. 1A). The parasite proteins were also resolved using 1-D electrophoresis comprising only one dimension i.e. SDS-PAGE. The required bands were taken out and electro-eluted (Fig. 1B) by a procedure described previously (11).

### Preparation of vaccines

The cocktail of 31+36+51 kDa was formulated with ALD (Autoclaved *L. donovani*), saponin and cationic liposomes as adjuvants. The ALD antigen was prepared by method (11).

In order to formulate the antigens with ALD as an adjuvant, 250μg of eluted proteins (Nanodrop was used to estimate protein concentration) were mixed with 2.5 mg of ALD antigen. The cocktail was also formulated with saponin as an adjuvant. Saponin was used at a dosage of 100 μg/mice (12).The positively charged liposomes were formulated using the commercially available kit (Sigma, USA). Sixty-three micromoles of phosphatidylcholine, 9 μmol ofcholesterol and 18μmol of stearylamine in the ratio of (7:1:2) were used. The antigen was entrapped by the method (13). In addition, the cocktail of 31+36+51kDa without any adjuvant was also used to immunize the animals.

### Immunization and challenge infection

Mice were categorized into different groups for experimental purpose. Group 1 comprised of normal control mice, group 2 were infected with 1×10^7^ promastigotes of *L. donovani* intravenously. Ten micrograms of cocktail antigens along with different adjuvants i.e. 31+36+51kDa; 31+36+51+ALD; 31+36+51+saponin and 31+36+51+liposome were used to immunize animals. The animals were immunized subcutaneously. After two weeks of final booster (2 boosters were given), animals were challenged with 1×10^7^ promastigotes. Different parameters were evaluated at 15 d post immunization and 30, 60 and 90 post-challenge days.

### Assessment of infection

Mice were sacrificed after 15 d post immunization and on 30, 60, 90 post challenge/ post infection days. The parasite load was counted by examining Giemsa stained impression smears of liverand expressed in terms of Leishman Donovan Units (LDU) (14).

### Delayed-type hypersensitivity response

Two days before the day of sacrifice, 40 μL of leishmanin and PBS was injected (i.e.) in the right and left footpad of mice, respectively. After 48 h, the thickness of right and left footpad was measured through verniercalliper. The DTH response was calculated by previously prescribed method (15).

### Assessment of antibody response

Serum specific IgG1 and IgG2a levels were assessed by conventional enzyme-linked immunosorbent assay (ELISA) by the method (16).

### Cytokine assays

Th1 and Th2 cytokine levels were assessed in frozen serum samples (−70°C) using commercially available kits from Diaclone, France.

### Statistical analysis

The statistical analysis was done by one-way ANOVA using SPSS software (ver.16, (Chicago, IL, USA).

### Ethics statement

The ethical clearance for conducting these experiments was taken from the Institutional Animal Ethics Committee of Panjab University, Chandigarh (Approval number-IAEC/284-295/3.9.12).

## Results

### DTH levels

**Fig. 2:**
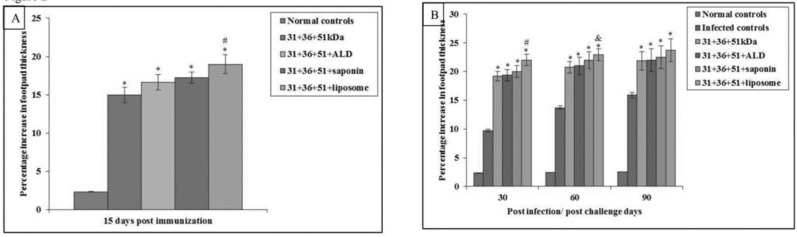
**A)** Percentage increase in footpad thickness (DTH response) in normal and immunized mice on 15 d post immunization. The data is presented as mean±S.D. of six mice per group. *P* value: Normal controls vs. 31+36+51kDa; 31+36+51+ALD; 31+36+51+saponin; 31+36+51+liposome. *(*P*<0.001) *P* value: 31+36+51 kDa vs. 31+36+51+ALD; 31+36+51+saponin; 31+36+51+liposome. #(*P*<0.001) **B)** Percentage increase in footpad thickness (DTH response) in infected and immunized mice on different post challenge days. The data is presented as mean±S.D. of six mice per group *P*-value: Infected controls vs. 31+36+51kDa; 31+36+51+ALD; 31+36+51+saponin; 31+36+51+liposome *(*P*<0.001) *P*-value: 31+36+51kDa vs. 31+36+51+ALD; 31+36+51+saponin; 31+36+51+liposome. #(*P*<0.001), &(*P*<0.05)

Immunization of mice with cocktail of three antigens i.e. 31 kDa, 36 kDa and 51 kDaalone and along with adjuvants ALD, saponin and liposome, elevated the percentage increase in footpad thickness (prechallenge) significantly in comparison to normal controls (*P*<0.001). When a comparison was made between animals immunized with only antigens i.e. 31+36+51kDa with other groups of animals, significantly increased DTH responses were seen in animals immunized with 31+36+51+liposome(*P*<0.001) (Fig.2A).

The DTH responses increased significantly in immunized animals as compared to the infected controls on all post challenge days (*P*<0.001). Addition of adjuvants i.e. ALD, saponin, and liposome further increased the DTH responses. Peak DTH responses were seen in mice immunized with cocktail of 31+36+51+liposome and the difference was significantly higher (*P*<0.001, 0.05) as compared to those immunized with cocktail of 31+36+51kDa except on day 90 (Fig.2B).

### Parasite load

**Fig. 3:**
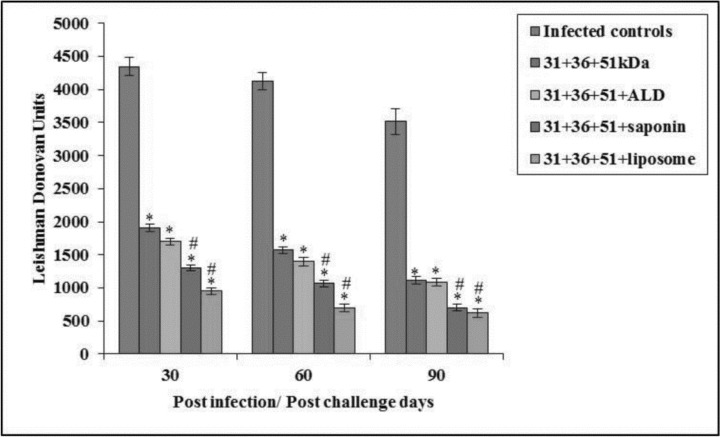
Parasite load in liver in terms of LDU in infected and immunized mice on different post challenge days. The data is presented as mean±S.D. of six mice per group *P*-value: Infected controls vs. 31+36+51kDa; 31+36+51+ALD; 31+36+51+saponin; 31+36+51+liposome *(*P*<0.001) *P* value: 31+36+51kDa vs. 31+36+51+ALD; 31+36+51+saponin; 31+36+51+liposome. #(*P*<0.001)

Parasite clearance was monitored in liver.The parasite load in immunized animals was significantly lesser as compared to the infected controls (*P*<0.001). The cocktail of 31+36+51+liposome imparted maximum protection with 78.16% to 82.37% reduction in LDU. In animals immunized with 31+36+51+saponin parasite load declined by 70.11% (30 post-challenge day) to 80.10% (90 post-challenge day). Further lesser amount of protection was conferred by immunization with 31+36+51+ALD with decline of parasite load by 69.01% on 90 post-challengedays. Least amount of protection was conferred by immunization with 31+36+51kDa showing 68.34% reduction on 90 post-challenge days as compared to the infected controls. When LDU of mice immunized with cocktails of 31+36+51+saponin and 31+36+51+liposome were compared to those immunized with 31+36+51kDa alone, significantly lower values were observed (*P*<0.001)(Fig.3).

### Cytokine responses Th1 cytokines

Immunization of mice with cocktail of 31+36+51kDa alone and along with adjuvants ALD, saponin, and liposome lead to significant increase in the pre-challenge IFN-γ and IL-12 levels as compared to normal controls (*P*<0.001)(Fig. 4A and 5A).

**Fig.4A:**
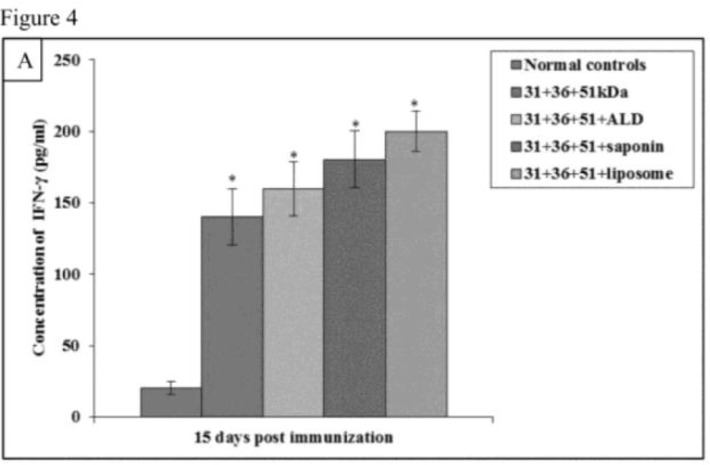
IFN-γ concentration in normal and immunized mice on 15 d post immunization. The data is presented as mean±S.D. of six mice per group *P*-value: Normal controls vs. 31+36+51kDa; 31+36+51+ALD; 31+36+51+saponin; 31+36+51+liposome. *(*P*<0.001) *P-*value: 31+36+51kDa vs. 31+36+51+ALD; 31+36+51+saponin; 31+36+51+liposome

**Fig.5A:**
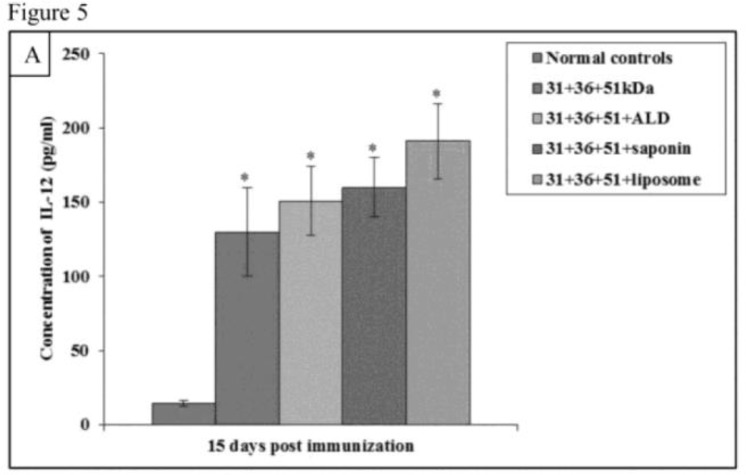
IL-12 concentration in normal and immunized mice on 15 d post immunization. The data is presented as mean±S.D. of six mice per group *P*-value: Normal controls vs. 31+36+51kDa; 31+36+51+ALD; 31+36+51+saponin; 31+36+51+liposome*P(<0.001) *P*-value: 31+36+51kDa vs. 31+36+51+ALD; 31+36+51+saponin; 31+36+51+liposome

The concentration of these cytokines was significantly higher in immunized animals (31+36+51+saponin and 31+36+51+liposome) as compared to the infected animals on all post challenge days (*P*<0.001) except IL-12 concentration in animals immunized with 31+36+51+saponin on 60 and 90post-challenge days. Maximum levels of Th1 cytokines was observed in animals immunized with 31+36+51+liposome. A significant increase was found in mice immunized with cocktail of 31+36+51+saponin and 31+36+51+liposome as compared to those immunized with 31+36+51kDa alone on all post challenge days (*P*<0.001, 0.05) except for IL-12 levels in animals immunized with cocktail of 31+36+51+saponin on day 60 and 90 as compared to those immunized with 31+36+51kDa alone (Fig.4B and 5B).

**Fig.4B:**
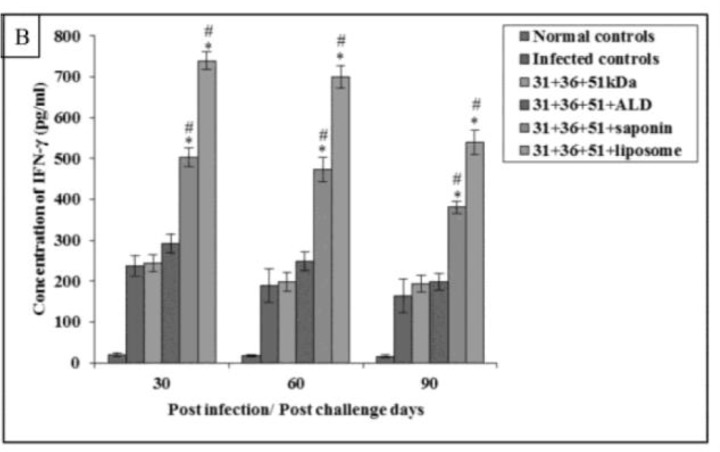
IFN-γconcentration in infected and immunized mice on different post challenge days. The data is presented as mean±S.D. of six mice per group *P-*value: Infected controls vs. 31+36+51kDa; 31+36+51+ALD; 31+36+51+saponin; 31+36+51+liposome. *(*P*<0.001) *P-*value: 31+36+51kDa vs. 31+36+51+ALD; 31+36+51+saponin; 31+36+51+liposome. #(*P*<0.001)

**Fig.5B:**
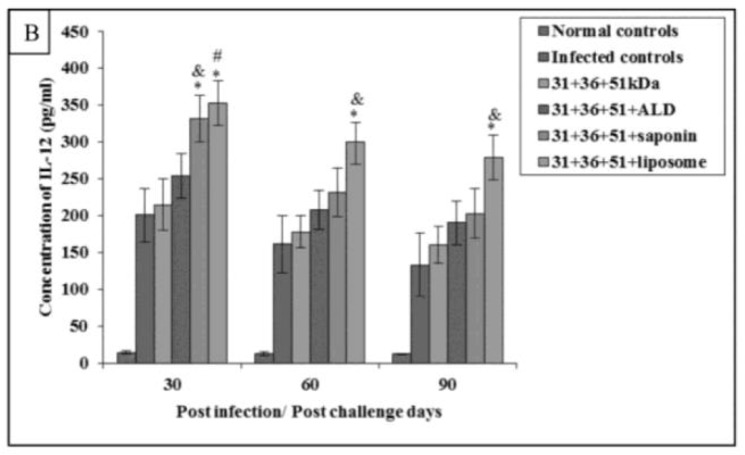
IL-12 concentration in infected and immunized mice on different post challenge days. The data is presented as mean±S.D. of six mice per group *P*-value: Infected controls vs. 31+36+51kDa; 31+36+51+ALD; 31+36+51+saponin; 31+36+51+liposome. *P (<0.001) *P* value: 31+36+51kDa vs. 31+36+51+ALD; 31+36+51+saponin; 31+36+51+liposome. #(*P*<0.001), & (*P*<0.05)

### Th2 cytokines

In animals immunized with cocktail of 31+36+51kDa alone and along with adjuvants ALD, saponin and liposome, the prechallenge IL-4 and IL-10 levels were significantly high as compared to normal controls (*P*<0.001) in all the groups (Fig.6A and 7A).

**Fig.6A:**
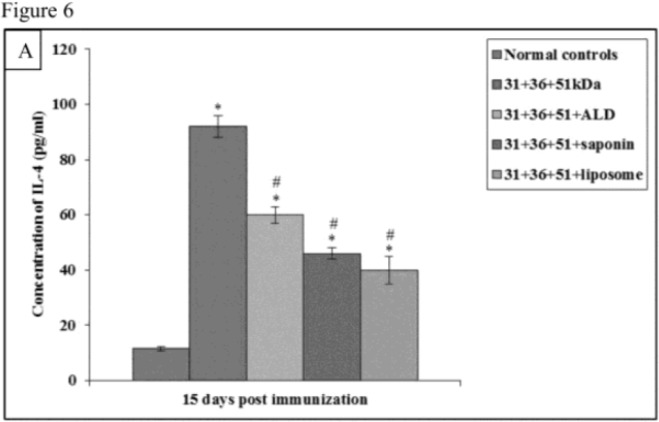
IL-4 concentration in normal and immunized mice on 15 d post immunization. The data is presented as mean±S.D. of six mice per group. *P*-value: Normal controls vs. 31+36+51kDa; 31+36+51+ALD; 31+36+51+saponin; 31+36+51+liposome. *(*P*<0.001). *P*-value: 31+36+51kDa vs. 31+36+51+ALD; 31+36+51+saponin; 31+36+51+liposome. #(*P*<0.001).

**Fig.7A:**
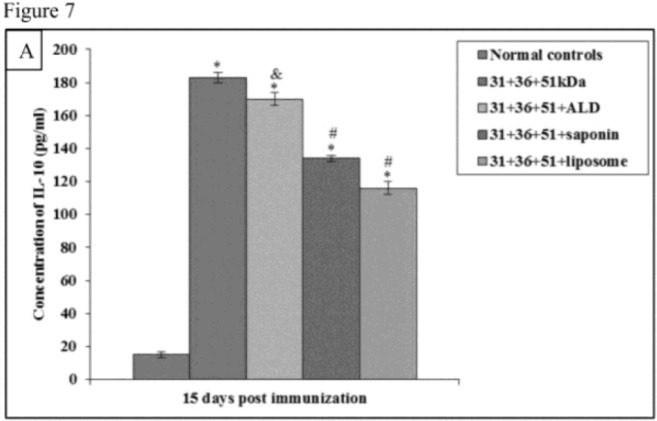
IL-10 concentration in normal and immunized mice on 15 d post immunization. The data is presented as mean±S.D. of six mice per group. *P*-value: Normal controls vs. 31+36+51kDa; 31+36+51+ALD; 31+36+51+saponin; 31+36+51+liposome. *(*P*<0.001) *P*-value: 31+36+51kDa vs. 31+36+51+ALD; 31+36+51+saponin; 31+36+51+liposome. #(*P*<0.001), &(*P*<0.05).

Immunized animals showed lesser concentration of IL-4 and IL-10 in comparison to the infected controls.

The concentration of IL-4 and IL-10 was significantly lower (*P*<0.001) in immunized animals as compared to the infected controls except for IL-10 levels in animals immunized with a cocktail of 31+36+51kDa on 30 post-challengedays. Maximum concentration of these cytokines was observed in animals immunized with 31+36+51kDa. Among immunized animals, minimum levels of this cytokine were observed in animals immunized with 31+36+51+liposome. A significant decrease (*P*<0.001, 0.05) in IL-4 andIL-10 levels was seen in animals immunized with cocktail of 31+36+51+ALD on 60 post-challengedaysand 31+36+51+saponin (except day 90) as compared to those immunized with 31+36+51kDa alone. Moreover, the levels of both the cytokines (IL-4 and IL-10) were also found to be significantly lower in mice immunized with cocktail of 31+36+51+liposome as compared to 31+36+51 kDa alone on all post challenge days (*P*<0.001, 0.05) (Fig.6B and 7B).

**Fig.6B:**
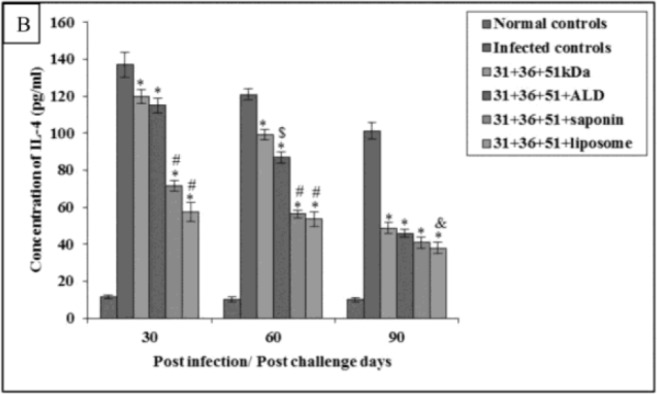
IL-4 concentration in infected and immunized mice on different post challenge days. The data is presented as mean±S.D. of six mice per group. *P*-value: Infected controls vs. 31+36+51kDa; 31+36+51+ALD; 31+36+51+saponin; 31+36+51+liposome. *(*P*<0.001), $(*P*<0.05) *P*-value: 31+36+51kDa vs. 31+36+51+ALD; 31+36+51+saponin; 31+36+51+liposome. #(*P*<0.001), &(*P*<0.05).

**Fig.7B:**
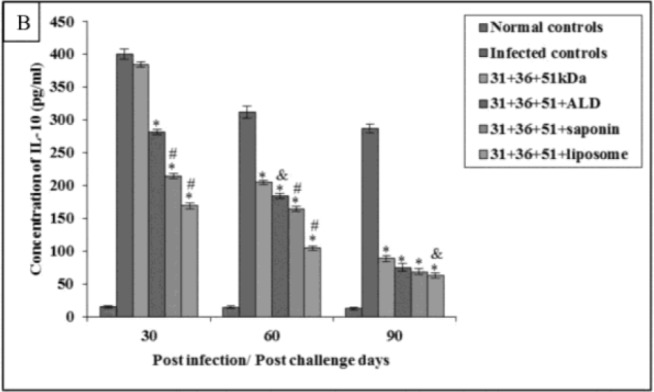
IL-10 concentration in infected and immunized mice on different post challenge days. The data is presented as mean±S.D. of six mice per group. *P*-value: Infected controls vs. 31+36+51kDa; 31+36+51+ALD; 31+36+51+saponin; 31+36+51+liposome. *(*P*<0.001). *P*-value: 31+36+51kDa vs. 31+36+51+ALD; 31+36+51+saponin; 31+36+51+liposome. #(*P*<0.001), &(*P*<0.05).

### Antibody Responses

The pre-challenge antibody levels were significantly high in all the immunized groups as compared to normal controls (*P*<0.001). When the animals immunized with only antigens i.e. 31+36+51kDa were compared with other groups of animals significantly decreased levels of IgG1 and increased levels of IgG2a were observed in animals immunized with 31+36+51+ALD, 31+36+51+saponin, and 31+36+51+liposome (*P*<0.001) (Fig.8A and 9A).

**Fig.8A:**
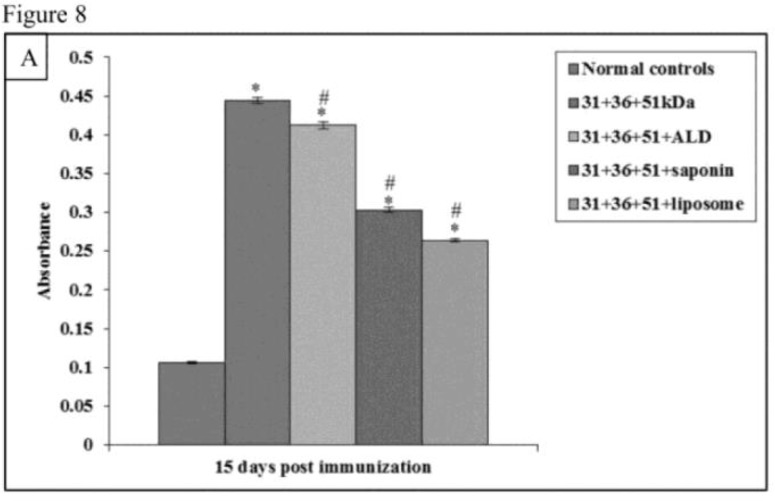
Levels of *Leishmania*-specific antibody (IgG1) in serum samples in normal and immunized mice on 15 d post immunization. The data is presented as mean±S.D. of six mice per group *P*-value: Normal controls vs. 31+36+51kDa; 31+36+51+ALD; 31+36+51+saponin; 31+36+51+liposome *(*P*<0.001). *P*-value: 31+36+51kDa vs. 31+36+51+ALD; 31+36+51+saponin; 31+36+51+liposome. #(*P*<0.001).

**Fig.9A:**
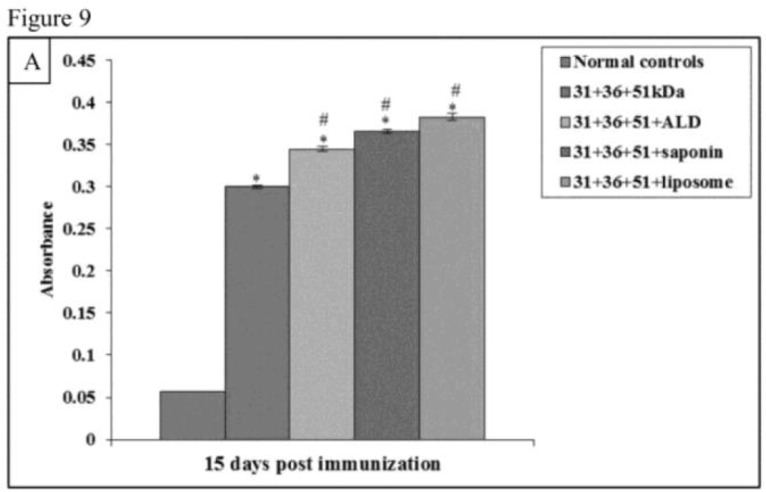
Levels of *Leishmania*-specific antibody (IgG2a) in serum samples in normal and immunized mice on 15 d post immunization. The data is presented as mean±S.D. of six mice per group *P*-value: Normal controls vs. 31+36+51kDa; 31+36+51+ALD; 31+36+51+saponin; 31+36+51+liposome.*(*P*<0.001) *P*-value: 31+36+51kDa vs. 31+36+51+ALD; 31+36+51+saponin; 31+36+51+liposome. #(*P*<0.001)

Maximum levels of IgG1 were obtained in infected controls group. Among immunized group maximum, IgG1 levels were obtained in animals immunized with 31+36+51kDa followed by groups of animals immunized with 31+36+51+ALD, 31+36+51+saponin, and 31+36+51+liposome. The IgG1 response in immunized animals was significantly lesser as compared to infected controls except in 31+36+51kDa and 31+36+51+ALD group on 60 and 90 post-challenge days. The IgG1 levels were also significantly lesser in mice immunized with 31+36+51+ALD (30 post-challenge day), 31+36+51+saponin and 31+36+51+liposome as compared to those immunized with 31+36+51kDa alone (*P*<0.001, 0.05) (Fig.8B).

**Fig.8B:**
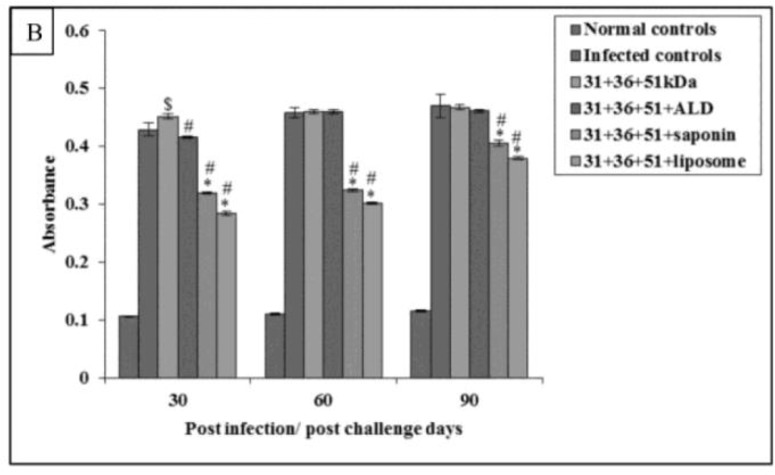
Levels of *Leishmania*-specific antibody (IgG1) in serum samples in infected and immunized mice on different post challenge days. The data is presented as mean±S.D. of six mice per group. *P*-value: Infected controls vs. 31+36+51kDa; 31+36+51+ALD; 31+36+51+saponin; 31+36+51+liposome. *(*P*<0.001), $(*P*<0.05) *P*-value: 31+36+51kDa vs. 31+36+51+ALD; 31+36+51+saponin; 31+36+51+liposome. #(*P*<0.001)

The maximum absorbance values of IgG2a were observed group of animals vaccinated with a cocktail of 31+36+51+liposome followed by those vaccinated with 31+36+51+saponin, 31+36+51+ALD, and 31+36+51kDa. The IgG2a levels were also significantly greater in mice immunized with 31+36+51+ALD, 31+36+51+saponin and 31+36+51+liposome as compared to those immunized with 31+36+51kDa alone (*P*<0.001) (Fig.9B).

**Fig.9B:**
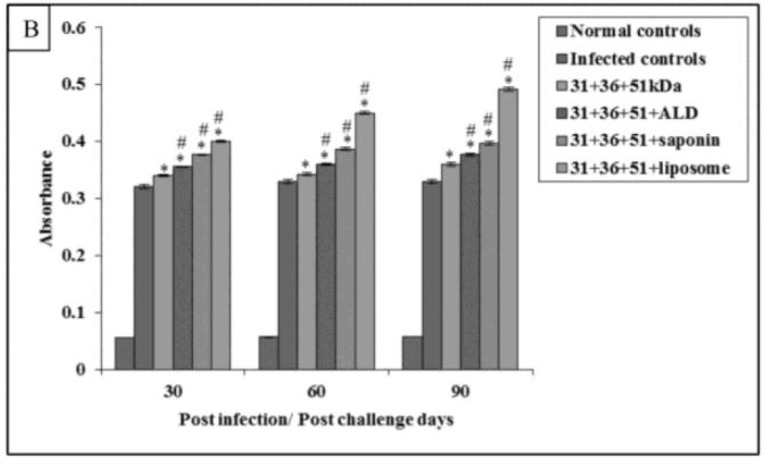
Levels of *Leishmania*-specific antibody (IgG2a) in serum samples in infected and immunized mice on different post challenge days. The data is presented as mean±S.D. of six mice per group *P*-value: Infected controls vs. 31+36+51kDa; 31+36+51+ALD; 31+36+51+saponin; 31+36+51+liposome. *(*P*<0.001) *P*-value: 31+36+51kDa vs. 31+36+51+ALD; 31+36+51+saponin; 31+36+51+liposome. #(*P*<0.001)

## Discussion

Among the tropical diseases, leishmaniasis is second in mortality and fourth in morbidity (17). The present scenario for vaccination is based on use of recombinant proteins (18). Different studies have been carried out with autoclaved *Leishmania major* and *L. donovani* antigens and encouraging results were observed in primates and murine models by many workers (19). Inoculation of killed vaccines leads to loss of infection-induced immunity in mice (20).

As concerns the first generation vaccines, single dose of alum-precipitated ALM along with BCG was found to be safe (21). Significant studies have also been reported by some workers in field of canine visceral leishmaniasis (22, 23). Some of the antigens included in the immunodominant 30- to 36-kDa fraction of *L. infantum* were detected by using proteomic approach (24).Hence, keeping in mind the significance of low molecular weight antigens, the present study has been designed.

To test novel therapeutic and immunoprophylactic agents, murine models of leishmaniasis have been used extensively (25). In our study mice were injected intravenously with 10^7^ promastigotes and sacrificed on various post challenge days. Maximum hepatic parasite load was seen in infected control group on 30 post-challenge days.

Thereafter, it was found to decline. It is in accordance with the earlier studies of *L. donovani* infection in BALB/c mice (26). In immunized animals, parasite load declined significantly as compared to infected controls. Maximum decline in parasite load i.e. 78.16% to 82.37% was found in group of animals immunized with a cocktail of 31+36+51+liposome. These results are in accordance with an earlier study in which formulation of single antigens (i.e. LD31, LD51, LD72, LD91) with liposome reduced parasite load by 74%, 72%, 65% and 46% respectively (27). Minimum reduction in parasite load was obtained when animals were immunized with a cocktail of 31+36+51kDa. In our study, when ALD was used as an adjuvant with a cocktail of 31+36+51kDa the percentage reduction of 60.91%–69.01% was achieved. These results are in line with a previous past study from our laboratory in which BALB/c mice immunized with a cocktail of Hsp70+Hsp 83+ALD induced 67%–91.90% decline in LDU as compared to the infected controls against murine VL (28). In the present study, maximum reduction in parasite load was obtained when liposome was used as an adjuvant as compared to saponin and ALD. Results are similar to a study (29) where mice inoculated with 2.5 μg of gp63 in liposomes reduced parasite load significantly.

Maximum DTH responses were observed in mice immunized with 31+36+51+liposome. Our results are in continuation with work (27) where mice immunized with liposomal LD31 and LD51 alone exhibited significant DTH responses (*P*<0.0001) as compared to control groups. In another study immunization of hamsters with proteins, P4-7 induced significantly higher levels of DTH responses on 45, 90 and 120 post-challenge days (30). Maximum DTH responses were observed with the use of liposome and it was followed by saponin and ALD. Results are similar with study (27) where mice immunized with liposomal LD31 and LD51 alone exhibited significant DTH responses. Our results are also similar to a study (31) in which mice immunized with rNH36 and FML along with saponin as adjuvant (100 μg) showed significant DTH responses.

In leishmaniasis, cellular immune responses play main role in recovery from infection. Th1 type of immune responses is crucial in preventing the growth of intracellular parasite like *Leishmania* (32). The antigen presenting cells pick up the leishmanial antigens (33). These cells activate specific T cells in the production of different cytokines and induce the macrophages to kill the parasites (33). IL-12 is a major immunoregulatory cytokine which initiates and maintain Th1 type of immune response and plays a significant role in the induction of IFN-γ production by T and NK cells (34).

In the present study, Th1 type of immune response was observed with elevated levels of Th1 cytokines. The best results were obtained with cocktail of 31+36+51+liposome. Our findings are in accordance with a study (35) in which vaccination of hamsters with cocktail of liposomal rCPA, rCPB and rCPC (2.5 μg each) plus MPL-TDM induced strong Th1 responses. According to the study (36), immunization of mice with Lip-rgp63-CpG ODN significantly raised the levels of IFN-γ. Our results are similar with study (37) where immunization of mice with LAg (leishmanial antigens) along with liposomes produced significantly high IFN-γ levels in comparison to the controls.

In our studies when mice were immunized using saponin as an adjuvant increased level of Th1 cytokines were observed as compared to infected controls. Decreased levels of IL-10 and IL-4 were reported on immunization of BALB/c mice with immunogenic preparations composed of *L. amazonensis* or *L. braziliensis* along with saponin against intravenous challenge with *L. chagasi* promastigotes (38). The present study is in concordance with the study (31) where immunization of BALB/c mice with NH36 DNA vaccine resulted in two to five-fold increase in IFN-γ producing CD4(+) T cells against leishmaniasis. As an adjuvant, liposome and saponins were more effective in producing Th1 specific cytokines as compared to ALD.

Appearance of anti-leishmanial antibodies in the sera of patients is another important characteristic feature of *Leishmania* infection. In comparison to infected controls, elevated levels of IgG2a were observed in the serum samples of immunized animals. In our study, maximum levels of IgG2a were observed in mice immunized with a cocktail of 31+36+51+liposome. Results of our study are in consistence with a study (39) where mice immunized with SLA+CpGs-ODN along with liposome showed stronger IgG2a antibody response. Similar results were reported from our laboratory in which elevated levels of IgG2a were observed in mice vaccinated with liposome-encapsulated 78kDa antigen as compared to infected control group (11). Promising results were also obtained when saponin was used as an adjuvant. Our results are similar to an earlier study in which immunization of mice with gp36 + saponin conferred protective immune response against visceral leishmaniasis with increased production of IgG2a (4).

From the present study, immunization of mice with a cocktail of 31+36+51 kDa (alone and along with various adjuvants) was protective and highly immunogenic against experimental visceral leishmaniasis. Best results were attained when liposome was used as an adjuvant.

## Conclusion

In the present situation development of either prophylactic or preventive vaccine has now become necessity to combat disease progression worldwide. Therefore, it is essential to identify new potential antigens. An effort has been made in this study to discover a poly-protein vaccine which when combined with suitable adjuvant confers long-lasting protection against the disease.
